# The acquisition and retention of ECG interpretation skills after a standardized web-based ECG tutorial–a randomised study

**DOI:** 10.1186/s12909-015-0319-0

**Published:** 2015-03-07

**Authors:** Signe Rolskov Bojsen, Sune Bernd Emil Werner Räder, Anders Gaardsdal Holst, Lars Kayser, Charlotte Ringsted, Jesper Hastrup Svendsen, Lars Konge

**Affiliations:** 1 Centre for Clinical Education, Rigshospitalet, Afsnit 5404, Teilumbygningen, Blegdamsvej 9, 2100 Copenhagen, Denmark; 2 Department of Cardiology, Hillerød Hospital, Dyrehavevej 29, 3400 Hillerød, Denmark; 3 Department of Cardiology, Rigshospitalet, Copenhagen University Hospital, Blegdamsvej 9, 2100 Copenhagen, Denmark; 4 Department of Public Health, Faculty of Health and Medical Sciences, University of Copenhagen, Øster Farimagsgade 5, opg, Q, Postboks 2099, CSS, 1014 Copenhagen, Denmark; 5 Faculty of Health, Aarhus University, Ndr. Ringgade 1, 8000 Aarhus C, Denmark

**Keywords:** Electrocardiogram (ECG), Electrocardiogram (ECG) interpretation, Retention of skills

## Abstract

**Background:**

Electrocardiogram (ECG) interpretation is of great importance for patient management. However, medical students frequently lack proficiency in ECG interpretation and rate their ECG training as inadequate.

Our aim was to examine the effect of a standalone web-based ECG tutorial and to assess the retention of skills using multiple follow-up intervals.

**Methods:**

203 medical students were included in the study. All participants completed a pre-test, an ECG tutorial, and a post-test. The participants were also randomised to complete a retention-test after short (2–4 weeks), medium (10–12 weeks), or long (18–20 weeks) follow-up.

Intragroup comparisons of test scores were done using paired-samples *t*-test. Intergroup comparisons of test scores were performed using independent-samples *t*-test and ANOVA, whereas demographic data were compared using ANOVA and Chi-squared test.

**Results:**

The overall mean test score improved significantly from 52.7 (SD 16.8) in the pre-test to 68.4 (SD 12.3) in the post-test (p < 0.001). Junior and senior students demonstrated significantly different baseline scores (45.5 vs. 57.8 points; p < 0.001), but showed comparable score gains (16.5 and 15.1 points, respectively; p = 0.48).

All three follow-up groups experienced a decrease in test score between post-test and retention-test: from 67.4 (SD 12.3) to 60.2 (SD 8.3) in the short follow-up group, from 71.4 (SD 12.0) to 60.8 (SD 8.9) in the medium follow-up group, and from 66.1 (SD 12.1) to 58.6 (SD 8.6) in the long follow-up group (p < 0.001 for all). However, there were no significant differences in mean retention-test score between the groups (p = 0.33). Both junior and senior students showed a decline in test score at follow-up (from 62.0 (SD 10.6) to 56.2 (SD 9.8) and from 72.9 (SD 11.4) to 62.5 (SD 6.6), respectively). When comparing the pre-test to retention-test delta scores, junior students had learned significantly more than senior students (junior students improved 10.7 points and senior students improved 4.7 points, p = 0.003).

**Conclusion:**

A standalone web-based ECG tutorial can be an effective means of teaching ECG interpretation skills to medical students. The newly acquired skills are, however, rapidly lost when the intervention is not repeated.

## Background

The electrocardiogram (ECG) is one of the most frequently used diagnostic procedures in medicine. By recording the electrical activity of the heart, the ECG can reveal cardiac abnormalities such as myocardial ischemia and arrhythmias – and the ECG can be the first or only indication of cardiac disease [1]. ECG interpretation is therefore of great importance for patient management [2-4], and all physicians who make clinical assessments based on ECGs should master this skill [5]. Basic ECG interpretation is taught as part of the undergraduate medical curriculum, and didactic lectures and teaching rounds are the predominant methods of instruction [6]. However, studies have reported a lack of proficiency in ECG interpretation as well as a perceived inadequacy of ECG training among medical students and young doctors [7-9]. This has prompted research on new methods to teach ECG interpretation skills to medical students.

The term *web-based learning* (WBL) is used to describe teaching interventions that use the Internet or a local intranet to deliver their educational content [10]. WBL has become increasingly popular in medical education for reasons such as flexibility in timing of participation and physical location of the learner and ease in updating the teaching material [10]. A meta-analysis of Internet-based learning for health professionals has shown that WBL appears to be as effective as traditional, non-Internet-based teaching methods with respect to knowledge, skills, behaviours in practice, and patient outcomes [11]. With regards to the ECG training of undergraduate medical students, it has been suggested that WBL can be superior to conventional teaching interventions when used as an adjunct to traditional teaching methods [12]. Also, in a recent study by Montassier et al., a standalone web-based ECG course was shown to be non-inferior to a traditional lecture-based course [13].

Many educational interventions are assessed by a post-test scheduled immediately after the course, whereas the retention of knowledge or skills over time is more rarely addressed. Some available retention studies use only a single follow-up interval [14]. Others test the same participants multiple times [15,16], which may itself enhance retention due to the so-called *testing effect* [17]. The present study is the first to examine the retention of ECG interpretation skills using multiple follow-up intervals in a study design that avoids the consequences of the testing effect.

Our aim was to explore (1) the effect of a standalone web-based ECG tutorial on the ECG interpretation skills of undergraduate medical students, and (2) the retention of skills following the tutorial using short, medium and long follow-up. The latter is explored amongst both junior and senior students in order to assess the effect of experience on retention.

## Methods

### Study design

**Figure 1:**
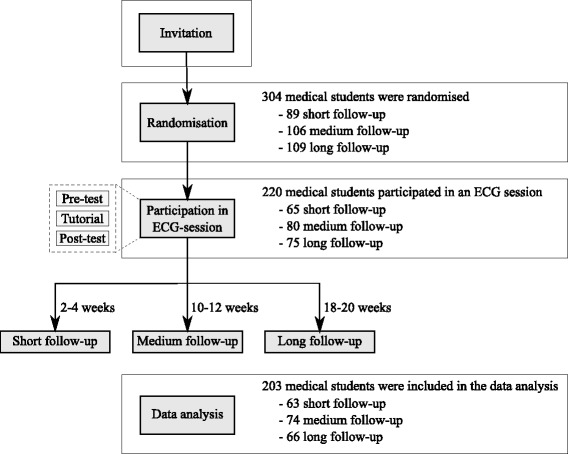
**Study design and flow of participants.** All students that responded to the study invitation were randomised and could participate in the ECG session. The ECG session consisted of a pre-test, the ECG tutorial and a post-test. The participants also completed a retention-test after either short, medium or long follow-up as determined by randomisation. The only difference between the three follow-up groups was the length of the follow-up interval. The number of participants in each step of the study is indicated in the right side of the figure.

In order to answer the first research question, all participants completed 1) a pre-test to establish ECG interpretation skills at baseline, 2) a web-based ECG tutorial, and 3) a post-test administered immediately following the tutorial to determine post-tutorial ECG interpretation skills. In order to examine the second research question, the participants were randomised to complete a retention-test after either short (2–4 weeks), medium (10–12 weeks), or long (18–20 weeks) follow-up. The study design is outlined in Figure 1.

### Power calculation

In order to detect a 5-point difference between the groups with an anticipated standard deviation of 10 points, we needed at least 63 participants in each follow-up group (alpha = 0.05 and power = 80%). To allow for uneven randomization and dropout, we aimed at including 240 participants.

### Participants

Medical students from the University of Copenhagen were invited to participate regardless of semester. An invitation was posted in the students’ magazine, and students were included on application. In order to ensure maximal follow-up, students were randomised to their follow-up groups before attending the tutorial, and they were not allowed to participate if they were unable to attend the retention-test (e.g. because of planned travel activities). Students were randomised using simple randomisation. The randomisation list was produced by an online random number service (www.random.org) and was administered by a person not otherwise involved in the study. The participants were not allowed to switch to another follow-up group.

All participants completed a questionnaire regarding gender, semester, age and interest in cardiology as a specialty. For data analysis purposes, students were then categorized as either junior or senior students, the former being students that had not participated in at least one lecture of the cardiorespiratory course on the 4th year. The participants were also surveyed about the number of ECGs interpreted during the past year, total time spend on ECG interpretation, and other relevant experiences.

### Ethical considerations

The Ethics Committee of the Capital Region of Denmark ruled that the study could be carried out without approval (protocol no. H-2-2014-071). All participants signed an informed consent.

### The ECG tutorial

The tutorial was developed by cardiologists from Copenhagen University Hospital with support from the e-learning unit at the Faculty of Health and Medical Sciences, University of Copenhagen.

The tutorial comprised (1) a theory module concerning the ECG and its components, the sinus rhythm, causes of arrhythmias including a detailed review of different arrhythmias, and theory on heart blocks, bundle branch blocks, hypertrophy patterns, heart axis, low voltage, and ischemia, (2) a training module with the opportunity to interpret 15 different ECGs (including clinical scenarios) with feedback, and (3) an ECG encyclopedia for use during the training module.

### The tests

Three ECG tests were developed; a pre-test, a post-test, and a retention-test. All tests had the same structure and all covered the same topics (e.g. arrhythmias, ischemia, etc.). Each test comprised 10 ECGs. No ECG appeared more than once, and none had been presented to the participants prior to the tests. For each ECG, the participants had to answer the same 10 questions resulting in a maximum total score of 100 points. The questions concerned the rhythm, PR-interval, QRS-complex, hypertrophy pattern, heart axis, low voltage, ST-elevations, ST-depressions, T-waves and Q-waves. The tests were designed using single-best answer multiple-choice questions with several distractors. Normally, two distractors are enough [18] but in order to mimic a clinical situation – where there are multiple possible diagnoses – a large number of possible answers (up to 23) were presented to the participants for each question.

### ECG sessions

Twenty ECG sessions, each with a maximum of 12 participants, were held at the Centre for Clinical Education during the fall of 2013. All participants booked the sessions themselves and were thus randomly distributed amongst the sessions without regard for their randomisation.

Each session (comprising the pre-test, the ECG tutorial, and the post-test) lasted 5 hours. After a 5-minute introduction, the participants had 40 minutes to complete the pre-test. The students then worked individually with the ECG tutorial for a total of 3 hours and 25 minutes including a 20-minute break. Following a 10-minute break, the participants then had to complete the post-test within 40 minutes. The students did not have access to the ECG tutorial during the tests. Sessions were monitored in order to resolve technical issues and to gather data on the participants’ use of the tutorial. No participants received personal teaching, as the intention was to simulate an individual, unsupervised training situation.

### Assessment of retention

A 40-minute retention-test was administered 2–4 weeks, 10–12 weeks and 18–20 weeks after the ECG session and the participants attended this according to their randomisation. The students did not have access to the tutorial between the session and the retention-test. All participants completed a questionnaire regarding the number of ECGs interpreted and time spend on ECG interpretation since their post-test. The retention-test was administered at the Centre for Clinical Education in order to ensure that the participants did not use any aids. In order to maximize the follow-up rate, the participants received the Danish version of the book ‘The ECG made easy’ on retention-test completion.

### Statistical analysis

Intragroup comparisons of test scores were done using paired-samples *t*-test. Intergroup comparisons of test scores were performed using independent-samples *t*-test and analysis of variance (ANOVA), whereas demographic data were compared using ANOVA (continuous variables) and Chi-squared test (categorical variables).

In all analyses, differences were considered statistically significant when the p-value was less than 0.05. Analyses were carried out using Statistical Package for the Social Sciences (SPSS), version 20.0 (IBM SPSS Statistics for Mac, Version 20.0. Armonk, NY: IBM Corp.).

## Results

A total of 304 medical students responded to the invitation and were randomised. 220 of these participated in an ECG session, however, 17 were excluded from the data analysis due to incomplete data sets (Figure 1). The demographic data of the remaining 203 participants are reported in Table 1. The three follow-up groups were comparable with regards to all surveyed demographics.

**Table 1:** **Baseline demographics of the 203 participants divided by follow-up groups**

	Short follow-up	Medium follow-up	Long follow-up	P-value
**Number of participants, N:**	63	74	66	
**Gender, N (%):**				
- Female	44 (69.8)	52 (70.3)	46 (69.7)	
- Male	19 (30.2)	22 (29.7)	20 (30.3)	0.997
**Junior and senior students, N (%):**				
- Junior*	27 (42.9)	30 (40.5)	27 (40.9)	
- Senior	36 (57.1)	44 (59.5)	39 (59.1)	0.959
**Age, mean (SD):**	26.2 (9.9)	24.4 (2.1)	24.7 (2.5)	0.157
Response rate:	62/63	74/74	66/66	
**No. of ECGs interpreted during the past**				
**year, mean (SD):**	14.6 (17.7)	23.9 (35.0)	20.4 (19.9)	0.120
Response rate:	61/63	73/74	64/66	
**Total time spend on ECG interpretation,**				
**mean hours (SD):**	9.7 (8.5)	13.7 (17.6)	12.6 (9.6)	0.194
Response rate:	61/63	74/74	65/66	
**Interest in cardiology as a specialty, N (%):**				
- Yes	20 (31.7)	26 (35.1)	20 (30.3)	
- No/do not know	42 (66.7)	46 (62.2)	45 (68.2)	0.789
Response rate:	62/63	72/74	65/66	
**Other relevant experiences, N (%):**				
- Clinical clerkship in cardiology	16 (25.4)	14 (18.9)	14 (21.2)	
- Other ECG or cardiology courses	8 (12.7)	14 (18.9)	11 (16.7)	
- Locum at a hospital	6 (9.5)	8 (10.8)	6 (9.1)	
- Research involving ECG interpr.	2 (3.2)	1 (1.4)	3 (4.5)	
- Teaches ECG interpretation etc.	1 (1.6)	1 (1.4)	0	
- Previously trained nurse etc.	1 (1.6)	0	1 (1.5)	
- Other	6 (9.5)	7 (9.5)	7 (10.6)	
- None	32 (50.8)	40 (54.1)	36 (54.5)	
Response rate:	63/63	74/74	65/66	

*Junior students are defined as students that have not participated in at least one lecture of the cardiorespiratory course on the 4th year.

**Figure 2:**
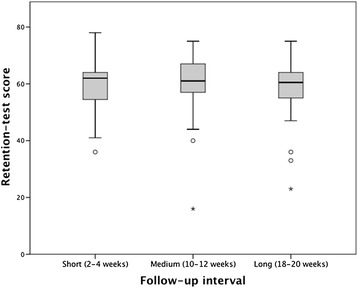
**Retention-test scores for the short, medium and long follow-up groups.** Box-plot representation of the retention-test scores for the short, medium and long follow-up groups. Each plot shows the median (centred vertical line), the middle 50% of the test scores (box), the highest and lowest score (top and bottom vertical lines) and outliers (dots and stars). As evident from the plot, there were no statistically significant differences in retention-test score between the three groups.

Test scores are presented in Table 2. We found a significant improvement in the overall mean test score from pre-test to post-test and a subsequent decrease between post-test and retention-test. The same trend applied when the test results were divided by follow-up groups; after an initial improvement in mean test score, each group experienced a decline in test score from post-test to retention-test with mean retention-test scores significantly higher than at baseline. However, there were no statistically significant differences in mean retention-test score between the three groups (p = 0.328; Figure 2).

**Table 2:** **Test scores, follow-up groups**

	Mean score (SD)			Mean Δ score pre-test → post-test	Mean Δ score post-test → retention-test	Mean Δ score pre-test → retention-test
	**Pre-test**	**Post-test**	**Retention-test**			
**Overall, N = 203**	52.7 (16.8)	68.4 (12.3)	59.9 (8.6)*	15.7 (p < 0.001)	−8.5 (p < 0.001)	7.2 (p < 0.001)
**Short follow-up, N = 63**	53.9 (15.9)	67.4 (12.3)	60.2 (8.3)	13.6 (p < 0.001)	−7.3 (p < 0.001)	6.3 (p < 0.001)
**Medium follow-up, N = 74**	53.6 (17.6)	71.4 (12.0)	60.8 (8.9)	17.8 (p < 0.001)	−10.6 (p < 0.001)	7.2 (p < 0.001)
**Long follow-up, N = 66**	50.6 (16.8)	66.1 (12.1)	58.6 (8.6)	15.4 (p < 0.001)	−7.4 (p < 0.001)	8.0 (p < 0.001)

*Pooled from follow-up intervals of all weeks (2–20 weeks).

The mean test scores and standard deviations of the pre-test, post-test and retention-test are reported for the overall number of participants and for each of the follow-up groups. The mean delta scores between pre-test and post-test, post-test and retention-test and pre-test and retention-test, respectively, are presented with associated p-values.

**Figure 3:**
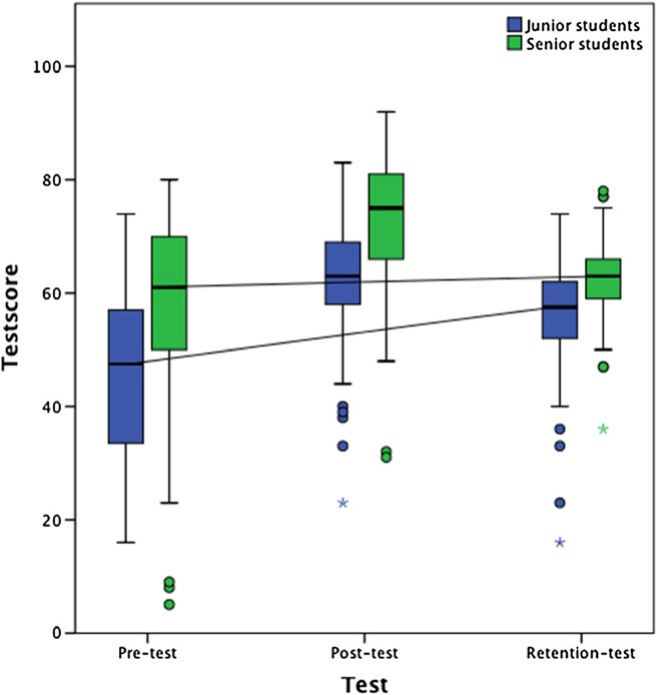
**Improvement in test score between pre-test and retention-test for junior and senior students.** Box-plot representation of the pre-test, post-test and retention-test scores for junior and senior students. Each plot shows the median (centred vertical line), the middle 50% of the test scores (box), the highest and lowest score (top and bottom vertical lines) and outliers (dots). The transverse lines that connect the pre-test and retention-test medians depict the improvement between pre-test and retention-test (pooled follow-up interval of 2–20 weeks). As evident from the plot, the overall improvement was greater among the junior students.

Table 3 shows the test results divided by junior and senior students. We found statistically significant differences between test scores at all test times with junior students consistently achieving lower scores (p < 0.001 in all three tests). Still, the overall time course was similar between the groups and was identical to the trend described above. In order to evaluate which of the two groups had benefitted most from the ECG tutorial by the end of the study, the delta scores between pre-test and retention-test were compared. Both junior and senior students had achieved statistically significant positive figures (10.7 points for junior students and 4.7 points for senior students); the delta score was, however, significantly greater among the junior students (p = 0.003). In Figure 3, the increase in test-score from pre-test to retention-test for both groups is visualized with a solid line.

**Table 3:** **Test scores, junior and senior students**

	Mean score (SD)			Mean Δ score pre-test → post-test	Mean Δ score post-test → retention-test	Mean Δ score pre-test → retention-test
	**Pre-test**	**Post-test**	Retention-test*			
**Junior students, N = 84**	45.5 (15.7)	62.0 (10.6)	56.2 (9.8)	16.5 (p < 0.001)	−5.8 (p < 0.001)	10.7 (p < 0.001)
**Senior students, N = 119**	57.8 (15.8)	72.9 (11.4)	62.5 (6.6)	15.1 (p < 0.001)	−10.5 (p < 0.001)	4.7 (p < 0.001)

*Pooled from follow-up intervals of all weeks (2–20 weeks).

The mean test scores and standard deviations of the pre-test, post-test and retention-test are reported for junior and senior students. The mean delta scores between pre-test and post-test, post-test and retention-test and pre-test and retention-test, respectively, are presented with associated p-values.

## Discussion

We found an effect of the web-based tutorial as the overall mean test score improved significantly between pre-test and post-test. This finding was expected since Internet-based learning in general has been shown to yield positive effects on outcomes such as knowledge and skills when compared to no intervention [11]. Our results also support the findings from two previous studies showing an effect of WBL used both alone and as part of a blended learning setting in the ECG training of medical students [12,13]. We observed that junior and senior students experienced comparable score gains suggesting that both groups benefited equally from the intervention. Thus, WBL can be an effective means of teaching ECG interpretation skills to medical students from a broad range of semesters.

The increase in overall test score found in this study is smaller than that reported by Montassier et al., who found a pre-test score of 9/20 points (45%) and a post-test score of 15.1/20 points (75.5%) [13]. Also, Fincher et al. detected an increase from 8.5% to 65.8% in their study of computer-assisted learning [19], the forerunner of WBL [20]. These differences in score gain may be due to differing ECG tutorials or scoring systems, but differences in study design must also be considered. In both of the aforementioned studies, the teaching interventions were available for the students for a total of 6 weeks, while the students in the present study only had access to the tutorial for a limited time on one occasion. This is important, since studies have shown that for equal amounts of study time, spaced practice (i.e. practice distributed over longer periods of time) can be superior to massed practice (i.e. bolus practice). This *spacing effect* has been demonstrated in areas as diverse as psychology [21], laparoscopic surgery [22] and the teaching of medical knowledge [23]. The current study design was chosen to standardize the intervention maximally. Studies have shown that some students never use an available WBL intervention [24] and that a discrepancy exists between self-reported use and actual use as assessed by user surveys and server statistics, respectively [25]. Our study design ensured that all students used the ECG tutorial and that the exact study time was known.

This study is the first to examine the retention of ECG interpretation skills among medical students using multiple follow-up intervals. Raupach et al. have previously investigated medium-term retention in two studies examining other ECG teaching interventions and learning incentives [26,27]. In both studies, retention-tests were administered 7–8 weeks after a cardiorespiratory module. Since we wanted to investigate the retention of ECG interpretation skills both before and after this medium-term follow-up, we decided on the current follow-up intervals.

We observed a rapid decline in ECG interpretation skills, as almost half of the initial gain was lost after 2–4 weeks. However, no additional loss had occurred at neither 10–12 nor 18–20 weeks. One explanation for this might be that the participants in the medium and long follow-up groups had maintained their skills during the longer follow-up periods. Indeed, ECG proficiency among residents has been found to correlate with level of training (i.e. postgraduate year) and interest in cardiology [8]. However, we found no correlation between mean score loss and ‘number of ECGs interpreted since post-test’ or ‘hours spend on ECG interpretation since post-test’ in the medium and long follow-up groups (data not shown).

The initial decline in interpretation skills had already occurred at the time of short follow-up, and the first part of the retention curve could not be examined. However, Bell et al. found a halving of the initial knowledge gain after just 3–8 days in their study of an online diabetes tutorial for resident physicians [28], which supports our findings.

The decrease in ECG interpretation skills observed at follow-up (calculated as percentage loss of initial knowledge gain) ranged from approximately 31-48% in the studies by Raupach et al. and approximately 53-60% in the current study. The trend towards greater score loss in our study could be due to several factors including the use of differing teaching interventions, learning incentives and scoring systems. Furthermore, studies have demonstrated improved retention rates when the initial learning intervention is spaced rather than massed [29,30]. As all teaching interventions administered by Raupach et al. were distributed over a six-week period, the spacing effect may have led to greater retention rates in these studies.

When examining the retention of ECG interpretation skills among junior and senior students, we found a significant decrease in both groups at the pooled follow-up of 2–20 weeks. The junior students had, however, retained significantly more than the senior students when comparing the retention-test score to the baseline score. No other study has compared the retention of ECG interpretation skills between experienced and non-experienced health professionals. However, Jensen et al. found that 6 months of clinical experience had a significant effect on the retention of Advanced Life Support (ALS) competencies [31] and Semeraro el al. found that anaesthesiology consultants retained newly acquired ALS-knowledge better than anaesthesiology interns [32]. Apposed to this study, both studies thus support the theory that pre-existing knowledge can lead to better retention. One explanation for our contradicting finding could be that the junior students improved due to the acquisition of a fundamental understanding of ECG interpretation, while the improvement among senior students was caused by the learning of more delicate facets of ECG interpretation, which are more rarely used and thus are not retained as well.

This study has several limitations. Firstly, the participants were included based on voluntary application. This may have affected the outcome, since volunteers has been shown to outperform non-volunteers [33]. The sampling approach did, however, result in a large study sample and this should be considered one of the study's strengths.

Secondly, in order to ensure maximal follow-up between post-test and retention-test, we chose to randomise the students at first contact. Accordingly, the dropout before actual study participation was relatively large. This was due, in some cases, to factors such as the timing of ECG-sessions or planned travel activities at the time of follow-up. However, the approach ensured a small in-study dropout rate and the final follow-up groups were comparable.

Thirdly, we observed that some participants needed time to familiarize with the tests. This lead to greater response rates in the post-test and retention-test, which in turn could have contributed to the score-gain observed between pre-test and post-test. However, the primary aim of this study was to examine the retention of ECG interpretation skills (i.e. the difference in test score between post-test and retention-test) and all participants were familiar with the test format when taking these tests.

Our findings on retention rates have important implications. Firstly, it is essential to acknowledge that although a standalone web-based tutorial may effectively impart ECG interpretation skills to medical students, this does not imply that these skills are retained in the long-term. Thus, educators should be aware that maintenance of newly acquired knowledge requires an effort from both teacher and student. Retention of knowledge can be increased through reinforcement, and factors such as the on-demand nature and the possibility of an infinite number of repetitions make WBL an effective reinforcing agent [34].

Secondly, although post-tests administered immediately after a teaching intervention may be used to assess the efficacy of a teaching intervention, they do not provide information on the long-term abilities of the students. If one wants to address this important matter, the post-test should be scheduled 2–4 weeks (or later) after the teaching intervention. This statement is supported by the findings obtained by Spitzer as early as 1939 [35]. Spitzer described a de-learning curve with a very rapid initial knowledge-loss and a subsequent steady state starting around 3 weeks and extending to at least 9 weeks.

Our results show that WBL can be used as a standalone intervention in the ECG training of medical students. However, exactly how WBL should be implemented into the medical curriculum in order to reach its maximum potential is not covered by the present study. In addition to using WBL as a standalone intervention, one might envision a setup where the WBL intervention is available to the students as an on-demand reinforcing agent following a traditional lecture-based intervention. Alternative combinations of teaching interventions might also prove valuable, yet further studies are needed to clarify which approach is the most effective.

Our results suggest that junior students may benefit more from a WBL intervention than senior students in terms of knowledge gain. However, busy work schedules during clinical rotations complicate the use of lecture-based ECG training. Hence, in practice WBL may prove to have the largest potential as a teaching tool for senior students and during residency.

## Conclusion

We found that a standalone web-based tutorial was an effective way to teach ECG interpretation skills to medical students from a broad range of semesters. The newly acquired skills were rapidly lost as the initial score gain was halved within 2–4 weeks, but we found no additional decrease in ECG interpretation skills at 10–12 weeks or 18–20 weeks.
